# EGFR T790M Mutation as a Possible Target for Immunotherapy; Identification of HLA-A*0201-Restricted T Cell Epitopes Derived from the EGFR T790M Mutation

**DOI:** 10.1371/journal.pone.0078389

**Published:** 2013-11-05

**Authors:** Teppei Yamada, Koichi Azuma, Emi Muta, Jintaek Kim, Shunichi Sugawara, Guang Lan Zhang, Satoko Matsueda, Yuri Kasama-Kawaguchi, Yuichi Yamashita, Takuto Yamashita, Kazuto Nishio, Kyogo Itoh, Tomoaki Hoshino, Tetsuro Sasada

**Affiliations:** 1 Department of Immunology and Immunotherapy, Kurume University School of Medicine, Kurume, Fukuoka, Japan; 2 Department of Surgery, Fukuoka University School of Medicine, Fukuoka, Fukuoka, Japan; 3 Division of Respirology, Neurology, and Rheumatology, Department of Internal Medicine, Kurume University School of Medicine, Kurume, Fukuoka, Japan; 4 Department of Pulmonary Medicine, Sendai Kousei Hospital, Sendai, Miyagi, Japan; 5 Metropolitan College, Boston University, Boston, Massachusetts, United States of America; 6 Cancer Vaccine Center, Dana-Farber Cancer Institute, Harvard Medical School, Boston, Massachusetts, United States of America; 7 Biostatistics Center, Kurume University Graduate School of Medicine, Kurume, Fukuoka, Japan; 8 Department of Genome Biology, Kinki University School of Medicine, Osaka-Sayama, Osaka, Japan; National Taiwan University, Taiwan

## Abstract

Treatment with epidermal growth factor receptor tyrosine kinase inhibitors (EGFR-TKIs), such as gefitinib and erlotinib, has achieved high clinical response rates in patients with non–small cell lung cancers (NSCLCs). However, over time, most tumors develop acquired resistance to EGFR-TKIs, which is associated with the secondary EGFR T790M resistance mutation in about half the cases. Currently there are no effective treatment options for patients with this resistance mutation. Here we identified two novel HLA-A*0201 (A2)-restricted T cell epitopes containing the mutated methionine residue of the EGFR T790M mutation, T790M-5 (MQLMPFGCLL) and T790M-7 (LIMQLMPFGCL), as potential targets for EGFR-TKI-resistant patients. When peripheral blood cells were repeatedly stimulated *in vitro* with these two peptides and assessed by antigen-specific IFN-γ secretion, T cell lines responsive to T790M-5 and T790M-7 were established in 5 of 6 (83%) and 3 of 6 (50%) healthy donors, respectively. Additionally, the T790M-5- and T790M-7-specific T cell lines displayed an MHC class I-restricted reactivity against NSCLC cell lines expressing both HLA-A2 and the T790M mutation. Interestingly, the NSCLC patients with antigen-specific T cell responses to these epitopes showed a significantly less frequency of EGFR-T790M mutation than those without them [1 of 7 (14%) vs 9 of 15 (60%); chi-squared test, p  =  0.0449], indicating the negative correlation between the immune responses to the EGFR-T790M-derived epitopes and the presence of EGFR-T790M mutation in NSCLC patients. This finding could possibly be explained by the hypothesis that immune responses to the mutated neo-antigens derived from T790M might prevent the emergence of tumor cell variants with the T790M resistance mutation in NSCLC patients during EGFR-TKI treatment. Together, our results suggest that the identified T cell epitopes might provide a novel immunotherapeutic approach for prevention and/or treatment of EGFR-TKI resistance with the secondary EGFR T790M resistance mutation in NSCLC patients.

## Introduction

Lung cancer is one of the most aggressive malignancies, but recently significant progress has been made in the therapeutic strategy against this disease [1]–[4]. In particular, epidermal growth factor receptor tyrosine kinase inhibitors (EGFR-TKIs), such as gefitinib and erlotinib, have been developed as a novel treatment option for patients with non–small cell lung cancers (NSCLCs) that possess somatic mutations in the tyrosine kinase domain of the epidermal growth factor receptor (EGFR) gene [5]–[7]. Prospective clinical trials of EGFR-TKI treatment in NSCLC patients with activating EGFR mutations, such as delE746-A750 (exon 19) and L858R (exon 21), have demonstrated high clinical response rates of approximately 80% [8]–[10]. Nevertheless, over time (median of 6–12 months), most tumors develop acquired resistance to EGFR-TKIs. Intense research into these NSCLCs has identified the secondary T790M mutation, which occurs in around 50% of patients with acquired resistance to EGFR-TKIs and is reported to negate the hypersensitivity of activating EGFR mutations [11]–[15]. However, there have been no effective treatment options for NSCLC patients with this secondary T790M resistance mutation.

In recent years, the field of cancer immunology has moved forward dramatically due to the identification of numerous tumor-associated antigens [16]–[18]. Notably, various approaches for therapeutic cancer immunotherapies have been developed and clinically examined, including cancer vaccines using tumor-associated proteins or peptides. Although the early-phase clinical trials demonstrated the feasibility and good toxicity profile of immunotherapeutic approaches, most of the late-phase randomized trials, with a few exceptions, failed to show beneficial therapeutic effects in patients compared to existing treatments [19], [20]. Such unexpected results might be attributed, at least in part, to the type of vaccine antigens employed for cancer immunotherapies. Currently, most vaccine antigens are derived from non-mutated self-antigens [21], which cannot be expected to show high immunogenicity due to the central and/or peripheral tolerance mechanism. In contrast, tumor-specific neo-antigens containing mutated amino acid sequences could be immunogenic, since they might be recognized as foreign by the host immune system [22], [23]. In particular, vaccine antigens derived from “driver mutations” might be an ideal target for immunotherapy, since they would rarely be lost from tumor cells via escape from immunological pressure [24]. Although there have been some reports demonstrating the feasibility of immunotherapies targeting mutated antigens [25]–[27], only a limited number of mutated antigens have so far been identified as potential targets for immunotherapies [24]. In NSCLCs, several T cell epitopes derived from mutated antigens were reported [28]–, but there have been no reports on the tumor-specific neo-antigens derived from EGFR driver mutations.

In the current study, we identified HLA-A*0201 (A2)-restricted antigenic T cell epitopes containing the mutated methionine residue of the EGFR T790M resistance mutation. Given their strong immunogenicity for human T cells, the identified T cell epitopes could provide a novel and promising immunotherapeutic approach for prevention and/or treatment of the secondary EGFR T790M mutation in NSCLC patients treated with EGFR-TKIs.

## Materials and Methods

### Peptides and cell lines

The peptides containing the wild-type (threonine) or mutated (methionine) residue at the position 790 of EGFR and control HLA-A2-restricted peptides, influenza M1_58-66_ (Flu-M1, GILGFVFTL) and HIV-derived epitope (SLYNTVATL), were provided by Thermo Fisher Scientific GmbH (Bremen, Germany) at purities of higher than 90%. T2 cells and NSCLC cell lines, NCI-H1975 (HLA-A2^−^ T790M^+^) and HCC827 (HLA-A2^−^ T790M^−^), were obtained from the American Type Culture Collection (ATCC; Manassas, VA). PC9 (HLA-A2^+^ T790M^−^), PC9/ZD (HLA-A2^+^ T790M^+^), 11–18 (HLA-A2^+^ T790M^−^), and YM-21 (HLA-A2^+^ T790M^−^) were obtained as described previously [33]–[36]. PC9/ZD cells were established as a gefitinib-resistant clone from PC9 cells [33], and were shown to harbor the T790M mutation of EGFR [34]. NCI-H1975-A2 cells were established by stably transfecting HLA-A2-negative NCI-H1975 cells with the plasmid carrying HLA-A*0201 cDNA (pCMV-HLA-A*0201) (data not shown). These cell lines were maintained in RPMI 1640 medium (Life Technologies, Rockville, MD) supplemented with 10% heat inactivated fetal calf serum (FCS) (MP Biologicals, Solon, OH), 100 µg/ml streptomycin (Life Technologies), and 100 IU/ml penicillin (Life Technologies). Expression of HLA-A2 on their cell surface was examined by flow cytometry with anti-HLA-A2 mAb (BB7.2; BD Biosciences, San Jose, CA).

### Blood samples from NSCLC patients

Blood samples were obtained from 6 healthy donors and 22 NSCLC patients treated with EGFR-TKI, gefitinib or erlotinib, between March 2012 and February 2013, at Kurume University Hospital (Kurume, Fukuoka, Japan) or Sendai Kousei Hospital (Sendai, Miyagi, Japan). This study was approved by the Institutional Review Board of Kurume University and Sendai Kousei Hospital, and conforms to the provisions of the Declaration of Helsinki. All of the patients had the EGFR gene mutations in exon 19 (delE746-A750) or exon 21 (L858R), and had received or were receiving gefitinib or erlotinib for treatment against advanced diseases at the blood sampling. For analysis of EGFR gene mutations in exon 19 (delE746-A750) or exon 21 (L858R), the peptic nucleic acid-locked nucleic acid (PNA-LNA) polymerase chain reaction (PCR) clamp method was adopted, using protocols described previously [37].

The EGFR T790M mutation was examined in cell-free DNA obtained from plasma of the patients, since no biopsy specimens for DNA analysis could be obtained because of difficult accessibility of tumors during or after EGFR-TKI-treatment. Heparinized whole blood from patients was centrifuged for 10 min at 400 g. After centrifuging for 10 min at 2000 g again, plasma was transferred to a new tube and stored at –80°C until use. Cell-free DNA was isolated from 200 µl of thawed plasma and eluted in 60 µl extraction buffer by using QIAamp DNA Blood Mini kit (Qiagen, Hilden, Germany). The EGFR T790M mutation was detected by the droplet digital PCR (ddPCR) system (QX100™ Droplet Digital™ PCR system; Bio-Rad Laboratories, Hercules, CA) according to the manufacturer’s instructions [38]. Briefly, the PCR reaction mixture was assembled from a 2X ddPCR Supermix for Probes (Bio-Rad), primers and FAM-labeled probe, and cell-free DNA (8 µl) in a final volume of 20 µl. The primers and FAM-labeled probes for T790M mutation (EGFR mutant allele assay, Hs00000106_mu) and reference (EGFR reference assay, Hs00000173_rf) were purchased from Applied Biosystems (Taqman Mutation Detection Reference Assays, Life Technologies). The droplets were generated from the prepared PCR reaction mixture and droplet generator oil by using QX100 Droplet Generator (Bio-Rad). Thermal cycling conditions were 95°C X 10 min (1 cycle), 95°C X 30s and 65°C X 60s (40 cycles), 98°C X 10 min (1 cycle), and 4°C hold by C1000 Touch Thermal Cycler (Bio-Rad). Analysis of the ddPCR data was performed with QuantaSoft analysis software that accompanies QX100 Droplet Reader (Bio-Rad). A diluted DNA from NCI-H1975 (T790M^+^) and T2 cells (T790M^−^) was used as a positive and negative control for this assay, respectively. Samples were prepared in duplicate for each experiment, and repeated twice to confirm reproducibility.

Clinical responses to EGFR-TKIs were evaluated by computed tomography (CT) according to the Response Evaluation Criteria in Solid Tumors (RECIST). Responses were confirmed at least every 4 weeks (for partial response) or every 6 weeks (for stable disease). Among the 22 NSCLC patients, 8 patients were sensitive to EGFR-TKIs at the time of the blood sample collection. In contrast, 14 patients showed primary (n  =  2) or acquired (n  =  12) resistance to EGFR-TKIs at the blood sampling.

### Prediction of HLA-A2-binding peptides containing the mutated methionine residue of the EGFR T790M mutation

Recent benchmarking has shown that NetMHC 3.2 (http://www.cbs.dtu.dk/services/NetMHC) and BIMAS (http://www-bimas.cit.nih.gov/molbio/hla_bind) are among the best preforming systems for predicting HLA-A2 binding peptides [39]. Thus NetMHC 3.2 and BIMAS were employed to predict 9-mer or 10-mer HLA-A2 binding peptides from the EGFR-T790M. For prediction of 11-mer HLA-A2 binding peptides, only NetMHC 3.2 was used because BIMAS does not perform predictions on 11-mer peptides. The top ranking peptides by either or both of these prediction servers were selected for further evaluation.

### HLA class I stabilization assay

The actual binding of predicted peptides to HLA-A2 molecules was evaluated by an MHC class I stabilization assay with TAP-deficient RMA-S cells stably transfected with the HLA-A*0201 gene (RMA-S/A2), according to the previously reported method with some modifications [40]. Briefly, RMA-S/A2 cells were cultured for 18 hours at 26°C in 1 ml of RPMI 1640 medium in the presence of synthetic peptides (10 µg/ml) and β2-microglobulin (2 µg/ml; Cortex Biochem, San Leandro, CA). After being washed, the cells were cultured for 3 hours at 37°C, and then stained with anti-HLA-A2 mAb (BB7.2), followed by flow cytometry (FACSCanto II; BD Biosciences). Data were analyzed by using the Diva software package (BD Biosciences). The binding capability of each peptide to HLA-A2 molecules was evaluated by the increase in mean fluorescence intensity (MFI) of the HLA-A2 expression, as follows: MFI increase (%)  =  (MFI with a given peptide – MFI without peptides)/(MFI without peptides) X 100. The HLA-A2-restricted influenza M1_58-66_ epitope (Flu-M1) was used as a positive control.

### Generation of antigen-specific T cells

Peptide-specific T cell lines were generated according to the previously reported method with slight modifications [41]. In brief, peripheral blood was obtained from 6 HLA-A2^+^ healthy donors and 22 HLA-A2^+^ NSCLC patients after written informed consent. HLA-A2 positivity was confirmed by flow cytometry with anti-HLA-A2 mAb (BB7.2). Peripheral blood mononuclear cells (PBMCs) (1×10^5^ cells per well) purified by Ficoll-Paque Plus (GE Healthcare, Uppsala, Sweden) density centrifugation were incubated with 10 µg/ml of each peptide in 96 round well plates (Nunc, Roskilde, Denmark) in 200 µl of the culture medium containing 45% RPMI 1640, 45% AIM-V medium (Life Technologies), 10% FCS, IL-2 (20 IU/mL; AbD Serotec, Kidlington, UK), and 0.1 mM MEM nonessential amino-acid solution (Life Technologies). At every 3 or 4 days, half of the culture medium was removed and replaced by new medium containing the corresponding peptide (10 µg/ml) and IL-2 (20 IU/mL). After 14 days, the cells were used for interferon (IFN)-γ ELSPOT or cytotoxicity assays.

### Immune assays

For IFN-γ ELISPOT assay (MBL, Nagoya, Japan), the peptide-stimulated PBMCs (2×10^4^ cells/well) were cultured in duplicate for 18 hours at 37°C with T2 cells (1×10^4^ cells/well) loaded with or without the indicated doses of specific or control peptides in a 96-well ELISPOT plate (MultiScreen HTS; Millipore, Bedford, MA) coated with anti-human IFN-γ mAb. After the plates were washed, biotin-conjugated anti-human IFN-γ mAb, streptavidin-ALP, and BCIP/NBT substrate were added to develop the spots, in accordance with the manufacturer’s instructions. The numbers of spots were then counted by an ELISPOT reader (CTL-ImmunoSpot S5 Series; Cellular Technology, Ltd., Shaker Heights, OH). The peptide-stimulated PBMCs were also tested for their reactivity against tumor cell lines by IFN-γ ELISPOT assay. In some cases, CD8^+^ T cells were isolated using a CD8 Negative Isolation Kit (Miltenyi Biotec, Bergisch Gladbach, Germany) from the peptide-stimulated PBMCs. To examine the HLA class I restriction, 10 µg/ml of anti-HLA class I mAb (W6/32: mouse IgG2a) was added into wells at the initiation of the culture. As an isotype control, anti-HLA-DR mAb (L243: mouse IgG2a) was used.

Peptide-stimulated PBMCs were also tested for cytotoxicity against tumor cell lines by a standard 6-hour ^51^Cr-release assay (PerkinElmer, Waltham, MA) using a MicroBeta2 LumiJET microplate counter (PerkinElmer). Effector cells and ^51^Cr-labelled cells (2000 cells/well) were cultured in duplicate in 96 round well plates at the indicated effector/target ratios. The spontaneous release and maximal release were determined by the target cells cultured in medium without or with 1% Triton X-100 (Wako Pure Chemical Industries, Osaka, Japan), respectively. The specific lysis was calculated as follows: specific lysis (%)  =  [(test release – spontaneous release)/(maximal release - spontaneous release)] X 100. The means of duplicate samples were used for calculation.

### Statistical Analysis

The difference in proportion of the presence of EGFR-T790M mutation between NSCLC patients with and without EGFR-T790M-specific immune responses was statistically evaluated by the chi-squared test (SAS 9.3; SAS Institute Inc., Cary, NC).

## Results

### Prediction of HLA-A2-binding peptides containing the mutated methionine residue of the EGFR T790M mutation

The amino acid sequences (9 to 11-mer) were predicted to bind HLA-A2 molecule by NetMHC 3.2 and/or BIMAS servers. Eight peptides (10- or 11-mer) containing the mutated methionine residue at the position 790 of EGFR (T790M), which showed better scores by either or both of these prediction servers, were selected for further evaluation (Table 1). But 9-mer peptides did not show good scores by the prediction servers.

**Table 1:** HLA-A2-binding capability of the predicted EGFR T790M-derived peptides.

Peptide name	Amino acid sequence	NetMHC 3.2 ANN IC50 (nM)	BIMAS score	HLA-binding capability (%)^a^
T790M-1	VQLIMQLMPF	3705	0.109	0
T790M-2	QLIMQLMPFG	4054	0.943	0
T790M-3	LIMQLMPFGC	837	24.921	0
T790M-4	IMQLMPFGCL	925	6.478	0
T790M-5	MQLMPFGCLL	977	51.770	207.5
T790M-6	LTSTVQLIMQL	4187	NA^c^	0
T790M-7	LIMQLMPFGCL	530	NA	19.1
T790M-8	IMQLMPFGCLL	118	NA	57.0
WT-5^b^	TQLMPFGCLL	2578	30.453	158.2
WT-7^b^	LITQLMPFGCL	5063	NA	0
Flu-M1	GILGFVFTL	18	550.927	234.4

a HLA-A2-binding capability was estimated by the increase in mean fluorescence intensity (MFI) as determined by flow cytometry after staining of RMA-S/A2 cells with anti-HLA-A2 mAb, as follows: MFI increase (%)  =  (MFI with a given peptide – MFI without peptides)/(MFI without peptides) X 100. The experiments were repeated three times and a representative result is shown.

b WT-5 and WT-7 peptides have wild-type sequences, corresponding to the T790M-5 and T790M-7 peptides, respectively.

c NA, not available.

### HLA-A2-binding capability of the EGFR-T790M-derived peptides

The HLA-A2-binding capability of the selected peptides was confirmed by cell surface HLA class I stabilization assay with the TAP-deficient cell line RMA-S, which stably expressed the HLA-A*0201 gene (RMA-S/A2). As illustrated in Table 1, three of the 8 selected peptides showed substantial binding to HLA-A2. Notably, the binding affinity of T790M-5 (MQLMPFGCLL) to HLA-A2 was much stronger than that of T790M-7 (LIMQLMPFGCL) or T790M-8 (IMQLMPFGCLL). The WT-5 peptide containing the wild-type threonine residue at position 790 of EGFR, which corresponded to T790M-5, showed high binding capability to HLA-A2, whereas the WT-7 peptide corresponding to T790M-7 did not bind to HLA-A2.

### Immunogenicity of the EGFR-T790M-derived HLA-A2-binding peptides in T cells from HLA-A2^+^ normal donors

**Figure 1:**
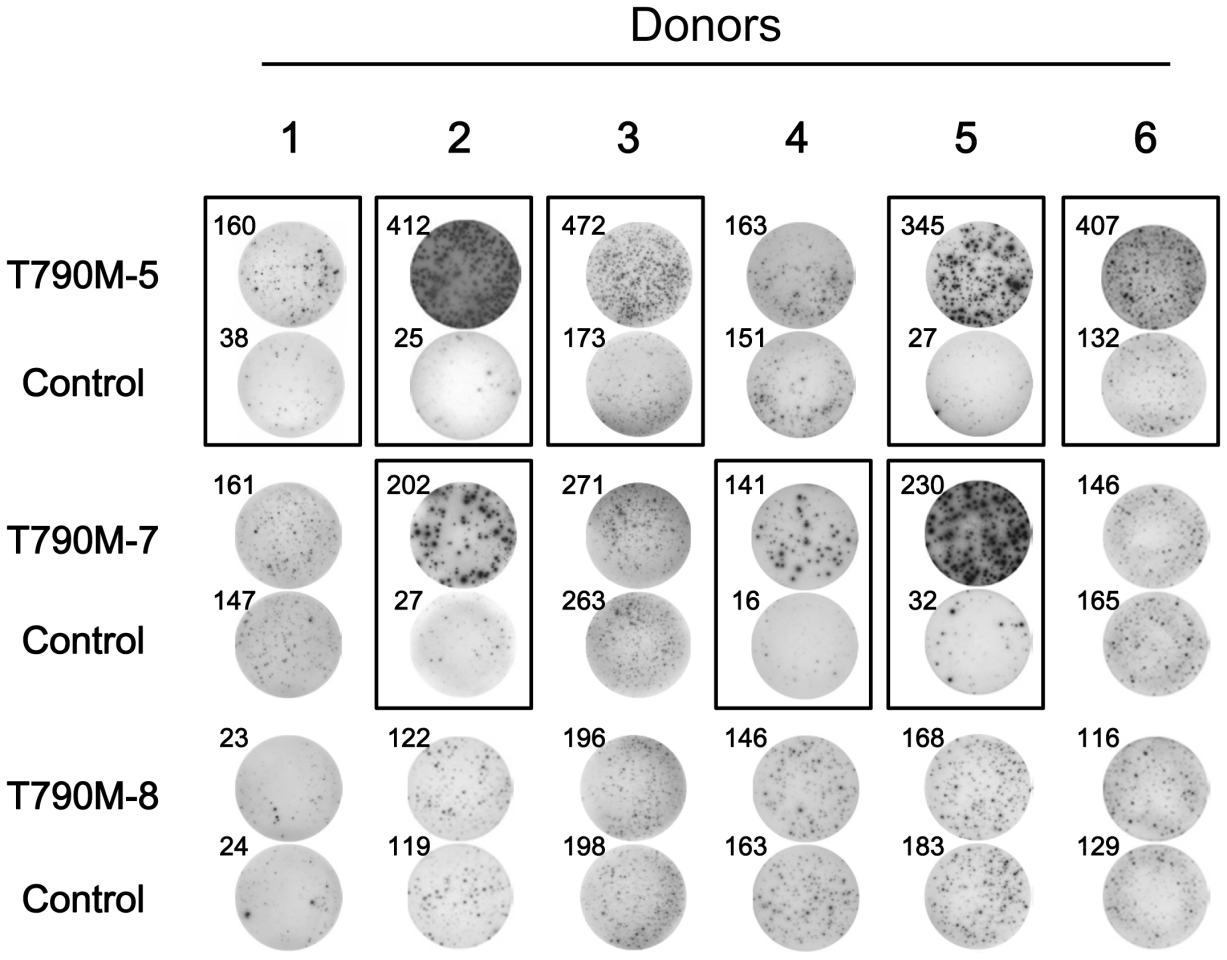
Immunogenicity of T790M-derived peptides in PBMCs from HLA-A2^+^ healthy donors. The immunogenicity of T790M-deived peptides, T790M-5, T790M-7, and T790M-8, was examined with PBMCs from 6 different HLA-A2^+^ healthy donors. PBMCs were stimulated 5 times with T790M-5, T790M-7, or T790M-8 peptides (10 µg/ml) every 3 or 4 days. The stimulated PBMCs (2×10^4^ cells/well) were examined for reactivity against T2 cells (1×10^4^ cells/well) pulsed with the corresponding peptides or control HIV peptide (10 µg/ml) by IFN-γ ELISPOT assay. The assays were carried out in duplicate wells, and representative wells in each donor are shown. The numbers of spots are shown for each well. Positive antigen-specific T cell responses are marked by closed boxes.

To examine the immunogenicity of the EGFR-T790M-derived HLA-A2-binding peptides, PBMCs from 6 different HLA-A2^+^ healthy donors were repeatedly stimulated with the synthetic peptides, T790M-5, T790M-7, or T790M-8. As shown in Figure 1, after repeated stimulation, T cell lines secreting IFN-γ in response to T790M-5 and T790M-7 could be established in 5 of 6 (83%) and 3 of 6 (50%) healthy donors, respectively. However, none of the 6 healthy donors showed antigen-specific T cell responses to T790M-8.

**Figure 2:**
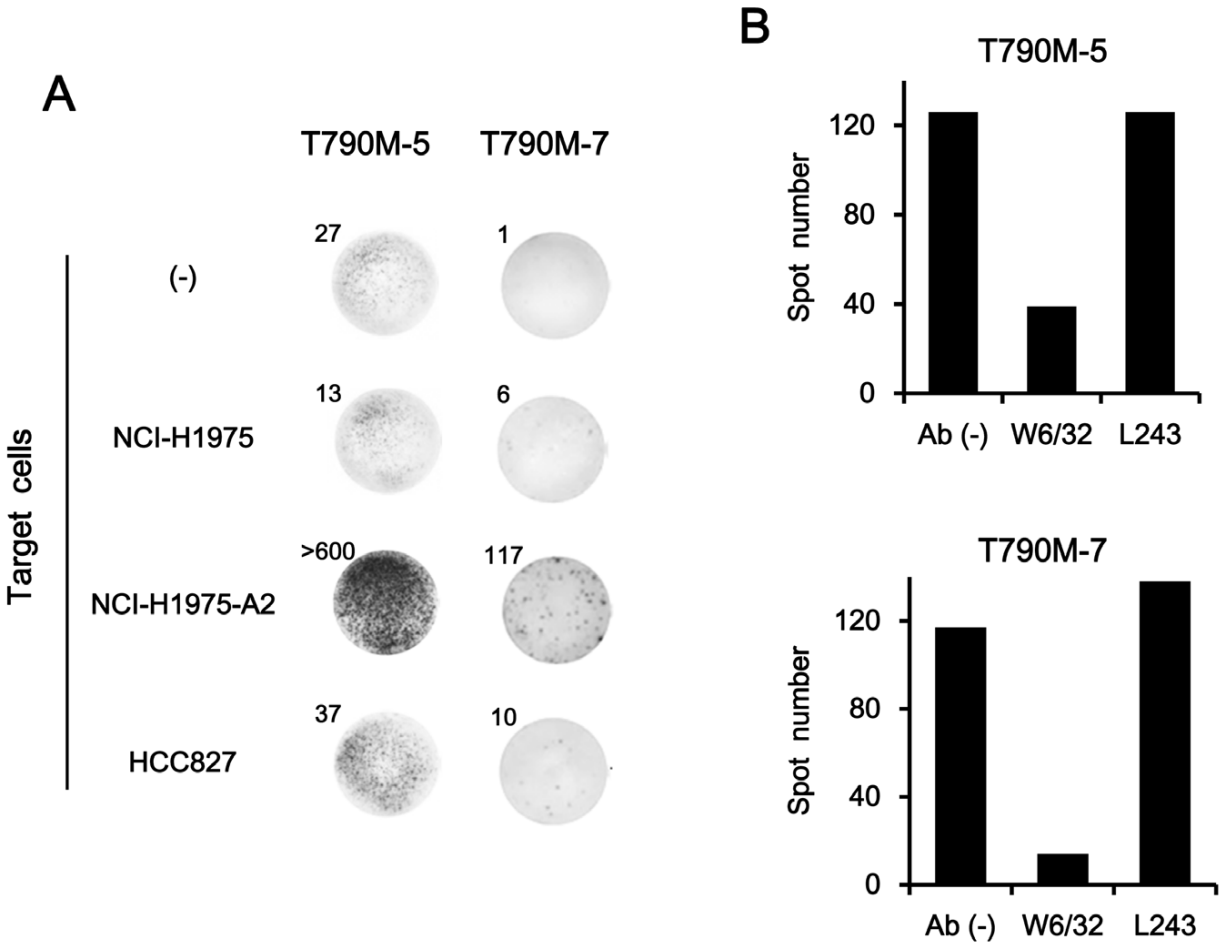
Reactivity of T790M-5- or T790M-7-stimulated T cells against HLA-A2^+^ NSCLC cells harboring EGFR T790M mutation. (A) CD8^+^ T cells (2×10^4^ cells/well) isolated from the T790M-5-stimulated PBMCs by magnetic beads were examined for their reactivity against different NSCLC cell lines (1×10^4^ cells/well), NCI-H1975 (HLA-A2^−^ T790M^+^), NCI-H1975-A2 (HLA-A2^+^ T790M^+^), or HCC827 (HLA-A2^−^ T790M^−^), by IFN-γ ELISPOT assay (left panel). The T790M-7-stimulated PBMCs (2×10^4^ cells/well) were similarly examined for their reactivity against the same tumor cell lines by IFN-γ ELISPOT assay (right panel). The assays were carried out in duplicate wells, and representative wells are shown. The numbers of spots are shown for each well. The experiments were repeated with blood from three different donors, and representative results are shown. (B) The T790M-5 and T790M-7-stimulated PBMCs (2×10^4^ cells/well) were examined for their reactivity against NCI-H1975-A2 cells (1×10^4^ cells/well) by IFN-γ ELISPOT assay in the absence or presence of 10 µg/ml of anti-HLA class I (W6/32) or anti-HLA-DR (L243) mAb. The assays were carried out in duplicate wells, and the means of spot numbers from duplicate wells are shown. The experiments were repeated with blood from three different donors, and a representative result is shown.

The T cell lines established after repeated stimulation of PBMCs with T790M-5 or T790M-7 were then examined for their reactivity against NSCLC cell lines, including NCI-H1975 (HLA-A2^−^ T790M^+^), NCI-H1975-A2 (HLA-A2^+^ T790M^+^), and HCC827 (HLA-A2^−^ T790M^−^), by IFN-γ ELISPOT assay. As shown in Figure 2A, the T cells lines established by T790M-5 or T790M-7 stimulation showed apparent IFN-γ production in response to NCI-H1975-A2, but not to HLA-A2-negative parental NCI-H1975. In addition, they showed no responses against an HLA-A2-negative, EGFR-T790M-negative cell line, HCC827. The IFN-γ secretion from T790M-5- and T790M-7-stimulated T cells in response to NCI-H1975-A2 was significantly inhibited by incubation with anti-HLA class I mAb (clone W6/32), but not by an isotype control mAb, anti-HLA-DR (clone L243), confirming that this response was MHC class I-restricted (Figure 2B).

**Figure 3:**
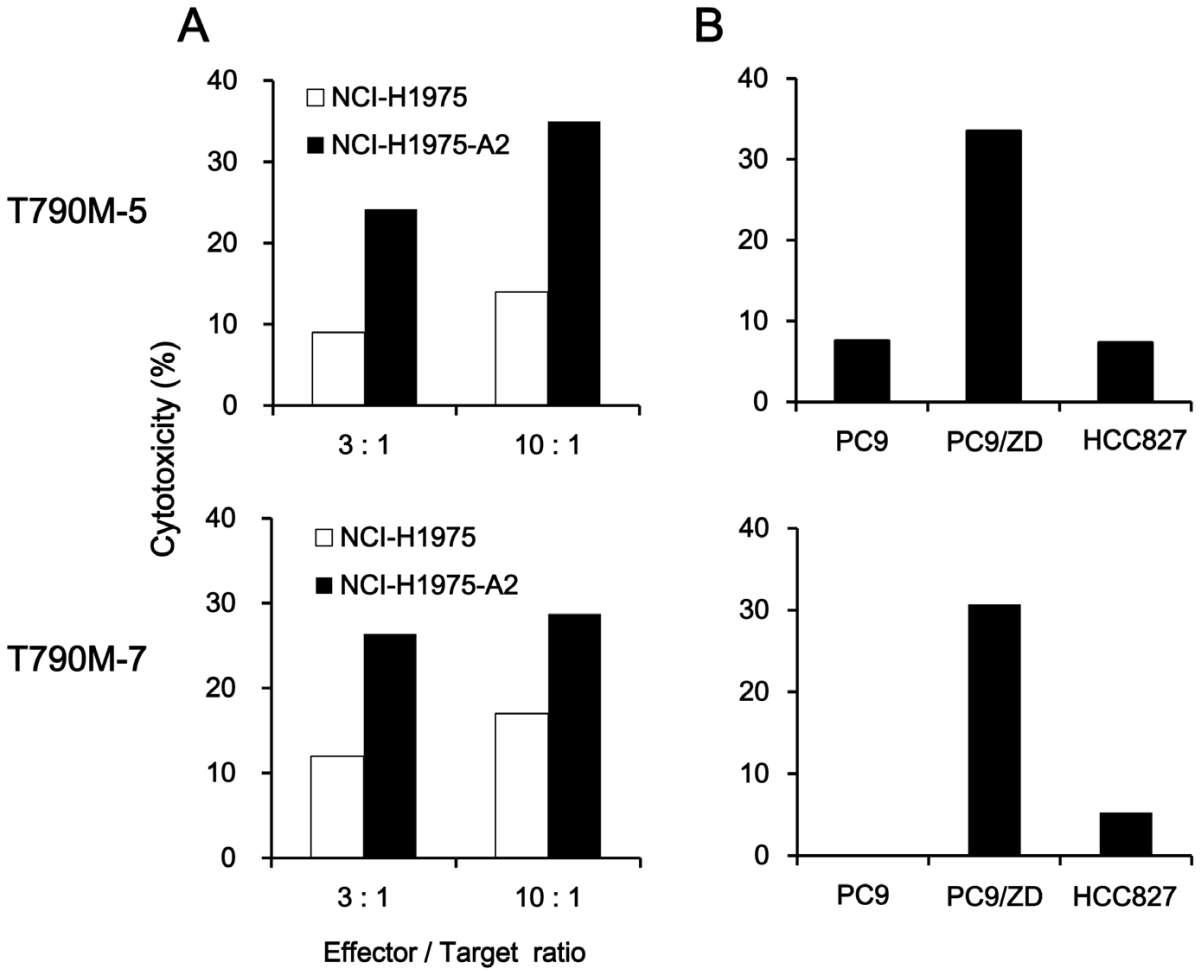
Cytotoxicity of the T790M-5- or T790M-7-stimulated T cells against NSCLC cells harboring EGFR T790M mutation. (A) The T790M-5- or T790M-7-stimulated PBMCs were examined for their cytotoxicity against the NSCLC cell lines (2×10^3^ cells/well), NCI-H1975 (HLA-A2^−^ T790M^+^) or NCI-H1975-A2 (HLA-A2^+^ T790M^+^), at the indicated effector/target ratios. (B) The T790M-5- or T790M-7-stimulated PBMCs (1×10^4^ cells/well) were examined for their cytotoxicity against the NSCLC cell lines (2×10^3^ cells/well), PC9 (HLA-A2^+^ T790M^−^), PC9/ZD (HLA-A2^+^ T790M^+^) or HCC827 (HLA-A2^−^ T790M^−^), at an effector/target ratio of 5∶1. The assays were carried out in duplicate, and the means of duplicate samples were used for calculation. The experiments were repeated with blood from three different donors, and a representative result is shown.

The reactivity of T cells against NSCLC cell lines was also investigated by ^51^Cr-release assay. Both T790M-5- and T790M-7-stimulated T cells showed higher cytotoxic activity against NCI-H1975-A2 than HLA-A2-negative NCI-H1975 (Figure 3A). Moreover, they demonstrated strong cytotoxic activity against HLA-A2-positive PC9/ZD cells harboring the EGFR T790M mutation (HLA-A2^+^ T790M^+^), but not against the original PC9 (HLA-A2^+^ T790M^−^) or HCC827 (HLA-A2^−^ T790M^−^) without the T790M mutation (Figure 3B). These results suggested that both T790M-5 and T790M-7 epitopes might be expressed on the surface of NSCLC cells harboring the EGFR T790M mutation in an HLA-A2-restricted manner.

### Cross-reactivity of T790M-5-stimulated T cells against the wild-type epitope or T790M-8

**Figure 4:**
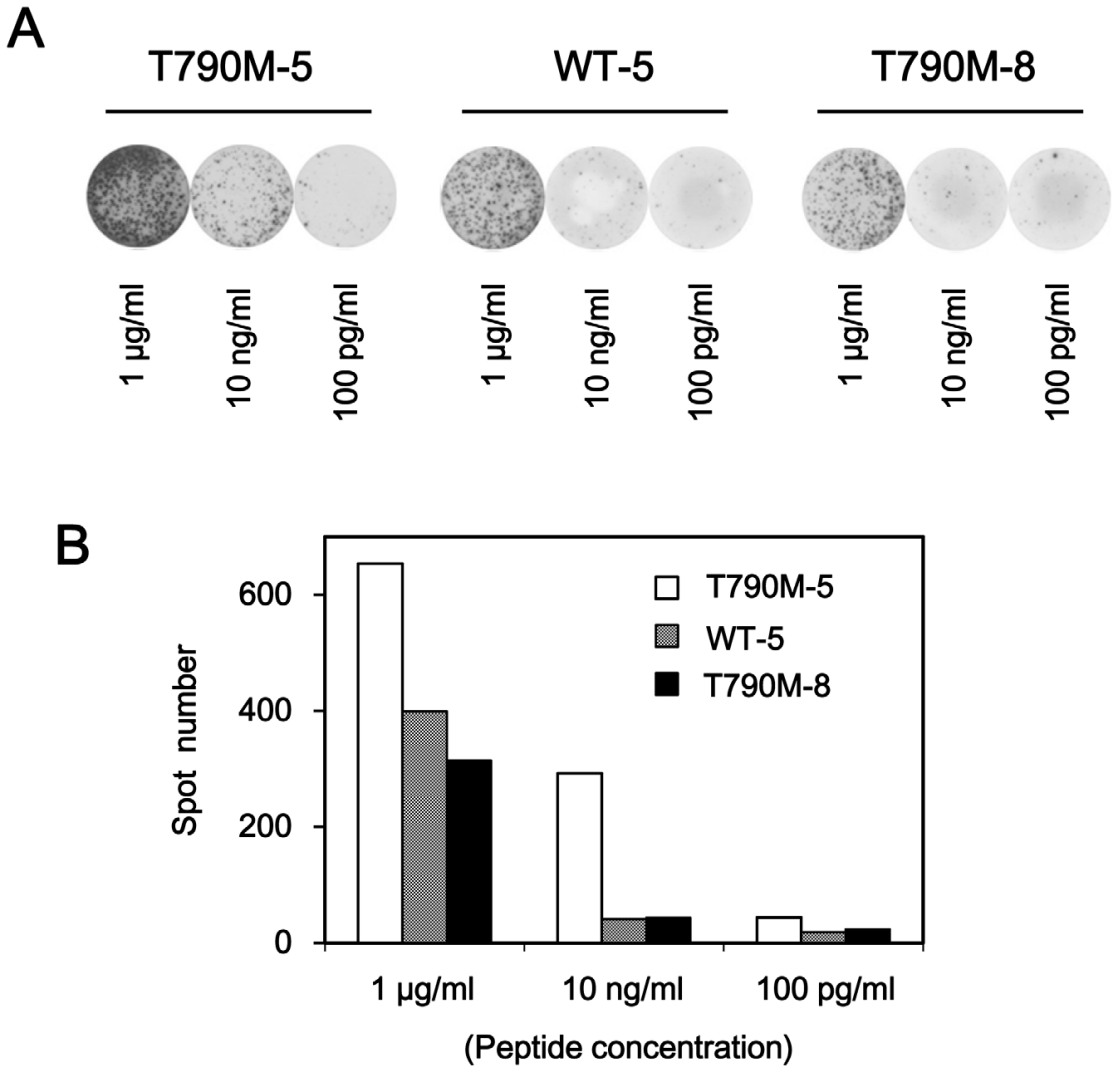
Cross-reactivity of T790M-5-stimulated T cells against the wild-type or T790M-8 peptide. The T790M-5 stimulated PBMCs (2×10^4^ cells/well) were examined for reactivity against T2 cells (1×10^4^ cells/well) pulsed with T790M-5, WT-5, or T790M-8 at the indicated concentrations by IFN-γ ELISPOT assay. The assays were carried out in duplicate wells. Representative wells (A) and the means of spot numbers from duplicate wells (B) are shown. The experiments were repeated with blood from two different donors, and a representative result is shown.

Since T790M-5 was different from the corresponding wild-type peptide, WT-5, by only a single amino acid sequence, we examined whether T cell lines established after repeated stimulation with T790M-5 could show reactivity against the corresponding wild-type peptide WT-5 (Figure 4). When the T790M-5-stimulated T cell lines were stimulated by a higher dose (1 µg/ml) of peptides for ELISPOT assay, the wild-type peptide WT-5 induced apparent, but weaker antigen-specific T cell responses, compared to the mutant peptide T790M-5. When a lower dose (10 ng/ml) of peptides was used for the assay, the T790M-5-stimulated T cell lines responded only to T790M-5, but not to WT-5. This finding suggested that the T cells established by stimulation with the mutant peptide T790M-5 possessed a substantial, but weaker (around 100 times less) cross-reactivity to the corresponding wild-type peptide. Similarly, the T cell lines established by T790M-5 stimulation possessed an apparent, but weaker (around 100 times less) cross-reactivity to the T790M-8 peptide, which has one additional amino acid extension at the N-terminal of T790M-5 (Figure 4). We did not examine the cross-reactivity of T790M-7-stimulated T cell lines, since the corresponding wild-type peptide, WT-7, could not bind to HLA-A2 (Table 1).

### Immunogenicity of the wild type HLA-A2-binding peptides in T cells from HLA-A2^+^ normal donors

**Figure 5:**
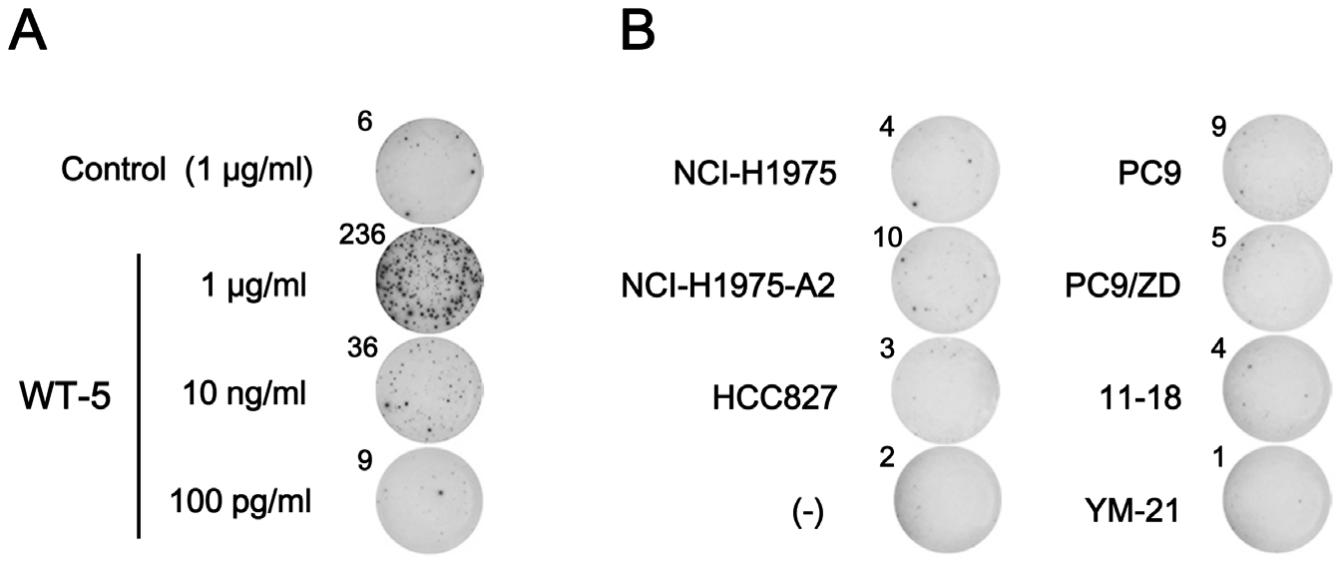
Reactivity of WT-5-stimulated T cells from an HLA-A2^+^ healthy donor. (A) PBMCs from an HLA-A2^+^ healthy donor were stimulated 5 times with WT-5 peptide (10 µg/ml) every 3 or 4 days. The WT-5-stimulated PBMCs (2×10^4^ cells/well) were examined for reactivity against T2 cells (1×10^4^ cells/well) pulsed with WT-5 or control HIV peptide at the indicated concentrations by IFN-γ ELISPOT assay. The assays were carried out in duplicate wells, and representative wells are shown. The numbers of spots are shown for each well. (B) The WT-5-stimulated PBMCs (2×10^4^ cells/well) were examined for their reactivity against different NSCLC cell lines (1×10^4^ cells/well), including NCI-H1975 (HLA-A2^−^ T790M^+^), NCI-H1975-A2 (HLA-A2^+^ T790M^+^), HCC827 (HLA-A2^−^ T790M^−^), PC9 (HLA-A2^+^ T790M^−^), PC9/ZD (HLA-A2^+^ T790M^+^), 11–18 (HLA-A2^+^ T790M^−^), and YM-21 (HLA-A2^+^ T790M^−^), by IFN-γ ELISPOT assay. The assays were carried out in duplicate wells, and representative wells with the spot numbers are shown. The data were from a single donor, since WT-5 was not immunogenic in 5 other healthy donors tested.

To examine the immunogenicity of the wild type HLA-A2-binding peptides, PBMCs from 6 different HLA-A2^+^ healthy donors were repeatedly stimulated with the synthetic peptides, WT-5 and WT-7. As shown in Figure 5A, after repeated stimulation, a T cell line secreting IFN-γ in response to WT-5 could be established in 1 of 6 (17%) healthy donors. However, none of the 6 donors showed antigen-specific T cell responses to WT-7 (data not shown), because this peptide could not bind to HLA-A2 (Table 1). When the WT-5-stimulated T cell line was stimulated by a higher dose (1 µg/ml) of WT-5 peptide for ELISPOT assay, it showed a strong antigen-specific response. In contrast, when a lower dose (10 ng/ml) of WT-5 peptide was used for the assay, the T cell line showed a much weaker response (Figure 5A), suggesting that the avidity of T cell receptor (TCR) of WT-5-stimulated T cells might be low.

The WT-5-stimulated T cell line was then examined for their reactivity against NSCLC cell lines, including NCI-H1975 (HLA-A2^−^ T790M^+^), NCI-H1975-A2 (HLA-A2^+^ T790M^+^), HCC827 (HLA-A2^−^ T790M^−^), PC9 (HLA-A2^+^ T790M^−^), PC9/ZD (HLA-A2^+^ T790M^+^), 11–18 (HLA-A2^+^ T790M^−^), and YM-21 (HLA-A2^+^ T790M^−^), by IFN-γ ELISPOT assay. As shown in Figure 5B, the WT-5-specific T cells showed reactivity against none of HLA-A2^−^ cell lines (HCC827 and NCI-H1975), HLA-A2^+^ T790M^+^ cell lines (NCI-H1975-A2 and PC9/ZD), or HLA-A2^+^ T790M^−^ cell lines (PC9, 11–18, and YM-21).

### Immunogenicity of T790M-5 and T790M-7 epitopes in T cells from HLA-A2^+^ NSCLC patients

We further investigated the immunogenicity of the T790M-5 and T790M-7 epitopes in 22 HLA-A2^+^ NSCLC patients harboring the EGFR gene mutations in exon 19 (delE746-A750) or exon 21 (L858R), who had been sensitive (n  =  8) or resistant (n  =  14) to the EGFR-TKI treatment at the blood sampling (Table 2). Among the 8 patients who had been sensitive to EGFR-TKIs, 4 patients (50%) showed antigen-specific T cell responses to T790M-5 or T790M-7. In contrast, only 3 of 14 EGFR-TKI-resistant patients (21%) revealed positive T cell responses to these epitopes. Interestingly, the NSCLC patients with antigen-specific T cell responses to these epitopes showed a significantly less frequency of EGFR-T790M mutation than those without them [1 of 7 (14%) vs 9 of 15 (60%); chi-squared test, p  =  0.0449], indicating the negative correlation between the immune responses to the EGFR-T790M-derived epitopes and presence of EGFR-T790M mutation in NSCLC patients. Of note, the T790M-5 and T790M-7-specific T cell lines established in these NSCLC patients were functional, since they showed substantial cytokine production or cytotoxicity against the NSCLC cell lines that harbored both the EGFR-T790M mutation and HLA-A2 (data not shown).

**Table 2:** Immunogenicity of T790M-5 and T790M-7 peptides in NSCLC patients treated with EGFR-TKIs.

				EGFR-TKI						
Patient	Age	Sex	Activating EGFR mutation	Primary response^a^	Resistance	Treatment period (days)	Timing of sampling (days)^c^	T790M-5^d^	T790M-7^d^	T790M mutation^e^
**EGFR-TKI-sensitive patients**										
1	59	M	Exon 19	PR	(–)	296^b^	-	89	102	(–)
2	60	M	Exon 19	PR	(–)	153^b^	-	0	0	(–)
3	77	F	Exon 19	PR	(–)	199^b^	-	50	0	(–)
4	65	F	Exon 19	PR	(–)	209^b^	-	0	0	(+)
5	64	F	Exon 19	PR	(–)	209^b^	-	0	0	(+)
6	71	F	Exon 19	PR	(–)	491^b^	-	0	64	(–)
7	67	F	Exon 21	PR	(–)	188^b^	-	68	0	(–)
8	72	F	Exon 21	SD	(–)	429^b^	-	0	0	(–)
**EGFR-TKI-resistant patients**										
1	60	F	Exon 21	PD	(+)	71	0	0	0	(+)
2	60	M	Exon 21	PD	(+)	51	94	0	0	(–)
3	81	M	Exon 19	SD	(+)	67	0	0	0	(–)
4	78	F	Exon 19	PR	(+)	642	0	0	0	(+)
5	57	F	Exon 19	PR	(+)	802	4	0	0	(+)
6	70	F	Exon 19	PR	(+)	395	152	0	26	(–)
7	64	M	Exon 19	PR	(+)	315	118	0	0	(+)
8	81	F	Exon 19	PR	(+)	457	0	0	0	(+)
9	76	F	Exon 21	PR	(+)	642	0	0	0	(+)
10	79	F	Exon 21	PR	(+)	350	76	0	0	(–)
11	74	M	Exon 21	PR	(+)	678	77	0	0	(–)
12	68	F	Exon 21	PR	(+)	499	26	157	0	(+)
13	59	M	Exon 21	PR	(+)	233	33	367	0	(–)
14	59	M	Exon 21	PR	(+)	230	78	0	0	(+)

Abbreviations: M, male; F, female; PR, partial response; SD, stable disease; PD, progressive disease.

a Primary clinical responses to EGFR-TKIs.

b Treatment with EGFR-TKIs was ongoing in the EGFR-TKI-sensitive patients at the time of blood sampling.

c The period (days) between discontinuance of EGFR-TKI-treatment and blood sampling.

d Spot numbers (/2×10^4^ cells) by ELISPOT assay.

e T790M mutation examined by droplet digital PCR in cell-free DNA from plasma.

## Discussion

The clinical benefit of EGFR-TKIs has been demonstrated in NSCLC patients with activating EGFR mutations [8]–[10], but most tumors develop acquired resistance via several different mechanisms, including the secondary T790M mutation that occurs in around 50% of patients with EGFR-TKI resistance [11]–[15]. However, there have been no effective treatment options for NSCLC patients with the secondary T790M resistance mutation. In the current study, we identified two HLA-A2-restricted cytotoxic T cell (CTL) epitopes containing the mutated methionine residue of the EGFR T790M mutation, T790M-5 and T790M-7. Given their high immunogenicity in human T cells, these epitopes might provide a novel immunotherapeutic approach for NSCLC patients with the T790M mutation. The limitation of the current study is that the T cell epitopes that we identified can be applicable only to NSCLC patients with HLA-A*0201. Although to increase the population coverage, we examined the immunogenicity of T cell epitopes restricted to other prevalent HLA-A types, including HLA-A*2402 and HLA-A*1101, we have not identified immunogenic epitopes restricted to these HLA-A types (data not shown). Nevertheless, HLA-A*0201 is one of the most prevalent HLA-A types in the world (http://www.pypop.org/popdata/2008/byfreq-A.php), and many of the peptides binding to HLA-A*0201 with high affinity have been shown to crossreact with other HLA-A2 supertype molecules, including HLA-A*0202, HLA-A*0203, HLA-A*0204, HLA-A*0205, HLA-A*0206, HLA-A*0207, HLA-A*6802, and HLA-A*6901 [42]. It would be of interest to determine whether the T790M-5 and T790M-7 epitopes can crossreact with other HLA-A2 supertype molecules.

For inducing effective anti-tumor immune responses, tumor antigens should be sufficiently immunogenic to stimulate antigen-specific T cells with high avidity. However, the T cell repertoires available for non-mutated “self-antigens” might have relatively low TCR avidity to them, since T cell reactivity against them can occur only when T cell tolerance is incomplete. In contrast, the “neo-antigens” derived from mutated amino acid sequences could be tumor-specific, and T cell repertoires with high TCR avidity to them might be available due to the lack of central and/or peripheral T cell tolerance [23], [24]. Indeed, in the current study, 5 of 6 healthy donors (83%) showed T cell responses to the mutated epitope T790M-5, whereas only 1 of 6 (17%) donors responded to the corresponding wild type epitope WT-5, suggesting that the immunogenicity of the wild type epitope may be low. Since WT-5-specific T cells with higher TCR avidity may be deleted by central and/or peripheral tolerance, only those with low TCR avidity could be activated. In fact, the WT-5-stimulated T cells could not react to HLA-A2^+^ NSCLC cells harboring wild-type EGFR. It may be possible that the TCR avidity of WT-5-stimulated T cells was too low to respond to the copy number of naturally processed WT-5 epitopes arrayed on HLA-A2^+^ NSCLC cells harboring wild-type EGFR.

In T790M-5 and T790M-7, the threonine residue at the 1^st^ and 3^rd^ positions of wild type epitopes is replaced by the mutated methionine residue, respectively. Although the amino acid residues at the 2^nd^ and C-terminal positions of epitopes have been known to act as the main anchoring sites in HLA-A*0201 [42], those at the 1^st^ or 3^rd^ positions of epitopes have also been reported to substantially affect the binding capability of peptides to HLA-A*0201 [43]–[47], which might modulate the immunogenicity [48]. Indeed, as shown in Table 1, the cell surface HLA class I stabilization assay demonstrated that T790M-5 and T790M-7 showed higher binding capability to HLA-A*0201 than the corresponding wild-type peptides, WT-5 and WT-7, respectively. In addition, side chains of amino acid residues at the 1^st^ position of some epitopes have been suggested to directly contact with TCRs and affect the interaction between the peptides and TCRs [49]. Therefore, the higher immunogenicity of T790M-5 and T790M-7 epitopes could possibly be explained by these mechanisms.

Recent analyses on T cell reactivity in cancer patients showed that a significant fraction of T cells recognized mutated neo-antigens, but not non-mutated self-antigens, in human cancers [22], [24]. In addition, T cell responses against mutated neo-antigens are expected not to show autoimmune toxicity against normal healthy tissues, suggesting that they might be a highly attractive target for immunotherapeutic manipulation. A large series of immunotherapy trials using non-mutated self-antigens demonstrated that clinical response rates were disappointingly low (around 3–5%) [19], but some studies have shown the potential benefit of targeting mutated neo-antigens [25]–[27]. For example, Sampson et al. demonstrated that peptide vaccines targeting EGFR variant III (EGFRvIII), which is a constitutively activated and immunogenic mutation not expressed in normal tissues but widely expressed in glioblastoma multiforme (GBM), were capable of inducing potent T- and B-cell immunity in GBM patients, and successfully eliminated tumor cells which expressed the targeted antigen, leading to an unexpectedly long survival time without any evidence of symptomatic collateral toxicity [25]. Weden et al. showed that a vaccine composed of synthetic long mutant K-RAS peptides induced long-term immune responses together with a potential clinical benefit in patients with pancreatic cancer after surgical resection [26]. Considering these feasible results of targeting mutated neo-antigens, cancer vaccines using the CTL epitopes derived from the T790M mutation, T790M-5 and T790M-7, might be promising for the treatment of NSCLC patients with this mutation. In particular, since the T790M mutation seems to be critical for acquired resistance to EGFR-TKIs [11]–[15], the selection of antigen loss variants as immune escape might be avoided during immunotherapy and tumor evolution.

Interestingly, the current study showed that the NSCLC patients with antigen-specific T cell responses to EGFR-T790M-derived epitopes showed a significantly less frequency of EGFR-T790M mutation than those without them, indicating the negative correlation between the immune responses to the EGFR-T790M-derived epitopes and presence of EGFR-T790M mutation in NSCLC patients. This finding could possibly be explained by the hypothesis that immune responses to the mutated neo-antigens derived from T790M might prevent the emergence of tumor cell variants with the T790M resistance mutation in NSCLC patients during EGFR-TKI treatment. Alternatively, it may be possible that the presence/occurrence of EGFR-T790M inhibits immune responses to the EGFR-T790M-derived epitopes. To further clarify the causal relationship between immune responses to the T790M-derived neo-antigens and the presence/occurrence of EGFR-T790M in NSCLC patients, both antigen-specific immune responses and T790M mutation status remain to be monitored at different time points (before, during, and after EGFR-TKI treatment) in the same patients in future study.

In the current study, T cells stimulated with T790M-5 showed cross-reactivity against the corresponding wild-type WT-5 peptide. However, it should be noted that the avidity of TCR to the WT-5 peptide in the T790M-5-stimulated T cells seemed to be around 100-times less than that to the T790M-5 peptide. Indeed, the T790M-5-stimulated T cells reacted to the PC9/ZD cells harboring the EGFR-T790M mutation, but not to the original PC9 cells expressing EGFR without this mutation. Based on this finding, the cross-reactivity of the T790M-5-stimulated T cells might not be high enough to induce autoimmune toxicity against normal tissues expressing wild-type EGFR.

In summary, we identified two novel HLA-A2-restricted, T790M-derived epitopes, which were highly immunogenic for human T cells. Our findings suggested that recognition of T790M-derived neo-antigens can occur in healthy donors and cancer patients, even in the absence of immunotherapy. Although more studies would be required to investigate whether immune responses to the identified epitopes are associated with clinical benefits and can be selectively enhanced by immunotherapies, the current study could provide important information on individualized immunotherapy for NSCLC patients.
